# Harnessing Nanoparticles for Immunomodulation and Vaccines

**DOI:** 10.3390/vaccines5010006

**Published:** 2017-02-14

**Authors:** Ariane C. Gomes, Mona Mohsen, Martin F. Bachmann

**Affiliations:** 1 The Jenner Institute, Oxford University, Old Road Campus Research Building, Roosevelt Drive, Oxford OX3 7DQ, UK; ariane.cruzgomes@ndm.ox.ac.uk (A.C.G.); mona.mohsen@kellogg.ox.ac.uk (M.M.); 2 Inselspital, Universitatsspital, Sahlihaus 1, 3010 Bern, Switzerland

**Keywords:** nanoparticles, virus-like particles, immunogen, vaccines

## Abstract

The first successful use of nanoparticles (NPs) for vaccination was reported almost 40 years ago with a virus-like particle-based vaccine against Hepatitis B. Since then, the term NP has been expanded to accommodate a large number of novel nano-sized particles engineered from a range of materials. The great interest in NPs is likely not only a result of the two successful vaccines against hepatitis B and Human Papilloma Virus (HPV) that use this technology, but also due to the versatility of those small-sized particles, as indicated by the wide range of applications reported so far, ranging from medicinal and cosmetics to purely technical applications. In this review, we will focus on the use of NPs, especially virus-like particles (VLPs), in the field of vaccines and will discuss their employment as vaccines, antigen display platforms, adjuvants and drug delivery systems.

## 1. Introduction

In the 18th century the English doctor Edward Jenner laid the foundation for a successful vaccination program against smallpox. Despite the rapid advancement in vaccine development since then, several drawbacks have been associated with these classical vaccines based on live, attenuated or chemically inactivated viruses. For attenuated vaccines, the most common adverse effects are the reactivation of the virulent state and severe side effects in subjects with weakened immune systems. Chemically inactivated vaccines are considered safer and easier to handle than attenuated vaccines, as they cannot regain their virulence. However, such vaccines stimulate a weaker immune response in general [1].

In the 1970s, scientists with safety concerns in mind started to harness the possibility of using subunit vaccines. Subunit vaccines consist of one or several specific proteins, which are either responsible for inducing neutralizing antibodies (e.g., hemagglutinin of influenza virus) or are causing disease themselves, such as for toxins (e.g., tetanus toxin). This type of vaccine has a similar safety profile when compared to inactivated pathogens, but, likewise, it does not induce life-long immunity and has low immunogenicity. The use of NPs provides an outstanding solution for this long-standing problem of safety versus immunogenicity, as nanoparticles (NPs)-based vaccines have shown, so far, the capacity to generate safer vaccines with an excellent immunological profile by giving recombinant subunit vaccines a viral finger-print [2,3,4]

NPs constitute a heterogeneous category of carriers that have in common their size, ranging from 1–1000 nm. NPs used in vaccines include virus-like particles (VLPs), viral vectors, lipid nanoparticles (LNPs), liposomes and cationic polymers [5], to name a few. The various classes of NPs used in other technical fields are extensively reviewed elsewhere [4,6]. Most used NPs for vaccines are derived from viral proteins, exhibit highly repetitive structures and form VLPs; other NPs, even if not derived from viruses, share some features such as size and repetitiveness.

Since the dawn of nanoparticle applications in vaccinology with the Hepatitis B vaccine, NPs have become an attractive display platform for antigens and for the delivery of adjuvants. The simplicity of production processes for nanoparticles and their high immunogenicity are major driving forces for using such a platform. The immunogenicity is largely due to the repetitiveness and polyvalence of the surfaces, which are a potent geometric pathogen-associated structural pattern (PASP) [7,8] capable of inducing a strong humoral response against the displayed antigen [9]. NPs in general can be assembled in vitro to encapsulate a variety of substances, which makes them a valuable tool to cargo immunomodulatory sequences such as siRNA, adjuvants such as immunostimulatory sequences (ISS) [5], and drugs such as antibiotics [5,10]. Thus, NPs can simultaneously display antigens of choice and package adjuvants as cargo, which further increases the possibilities to tailor the conditions and characteristics of the vaccine by modulating the immune response raised by such particles.

The size of NPs is arguably one of the most important features of their immunogenicity. Efficient draining of antigens from the periphery to the lymph nodes allows the simultaneous delivery of both NPs and their cargo to relevant populations of immune cells and their intracellular compartments which initiate the adaptive immune response. Nano-sized particles have been shown to freely drain to lymph nodes [11] and preferentially be taken up by lymph node–resident dendritic cells and macrophages [8,12], the two main antigen-presenting cells (APCs).

The inherent versatility of NPs for antigen display and packaging of immune-stimulatory substances allows their broad application in a large number of indications, going far beyond use as prophylactic vaccines against infectious diseases. Indeed, VLP-based strategies for active immunization against chronic diseases such as hypertension [13], nicotine addiction [14], arthritis [15] and Alzheimer′s disease [16,17] have been clinically assessed. An important feature of VLPs for these indications is their ability to overcome B cell tolerance to allow the induction of self-specific antibodies, targeting endogenous molecules such as angiotensin II for hypertension [13] or IL-1 for type II diabetes [18]. More details on NP-based vaccines in clinical trials are reviewed elsewhere [2]. In the following sections, we will cover the landscape of available NPs, the immunology behind nanoparticles, focusing on NPs used for vaccination programs, popular applications of NPs and recent developments in the field.

## 2. Categories of Nanoparticles

The range of NPs available for vaccination has expanded dramatically over the past 30 years [4]. Among the most popular NPs, at least seven broad categories can be identified. All of them share the repetitive structure, a size in the nanometer range, permissibility to functionalization of the surface and an adjuvant effect for vaccines. They differ, however, remarkably in size within the given nanometer range, the nature of their building blocks, the mode of antigen presentation—packaged within the NP, chemically fused to the surface, or genetically fused to the VLP—the modulatory outcome to the immune response and, finally, their potency as immunogens. The different types of NPs are summarized in Table 1.

Most of the NPs are built from organic, biological sources. One reason for this is likely the continuing concerns of toxicity due to persistence in the host of non-biodegradable NPs, such as gold [19] and carbon NPs [20]. Although inorganic NPs do hold advantages, such as consistent and controllable methods of synthesis and rigidity of structure, adverse events, sometimes of a serious nature, may be associated with these NPs. The nanometer size of any NPs allows access to usually protected compartments, such as the lower lungs. In these locations, inorganic NPs which are not easily degradable can cause persistent inflammation and ultimately fibrosis [21]. It is well appreciated, however, that the toxicity of NPs is not an exclusive feature of inorganic NPs, as long-term persistence is not the sole cause of toxic side effects, but also size-dependent bio-distribution through the system, the target cells, the charge of the surface and the hydrophobicity of such particles [22].

**Figure 1:**
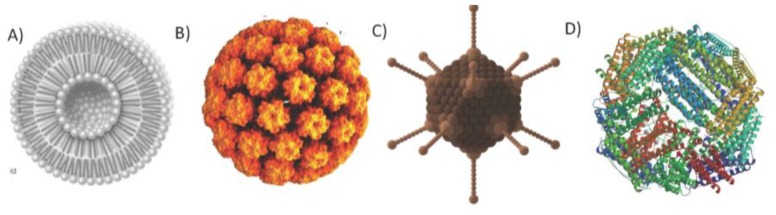
Nanoparticles. (**A**) Liposome: schematic representation of a phospholipidic liposome; (**B**) VLP derived from HPV virus, PDBID: 1DZL; (**C**) Viral vector: schematic representation of viral adenovirus.; (**D**) Self-assembled proteins: X-ray structure of ferritin PDBID: 2X17. Protein Data Bank Identification (PDBID).

Organic NPs, on the other hand, such as emulsions, immune-stimulating complexes (ISCOM), liposomes (Figure 1A) and protein-based NPs, can be better tolerated in general, as they are easily degraded. Emulsions are composed of a surfactant and a solvent, and have been used successfully as adjuvants in humans with acceptable safety profiles as seen with AS01 and AS03 from GlaxoSmithKline (GSK) in vaccines against malaria [23] and H1N1 [24], respectively. ISCOMs are immune-stimulating complexes formed by an antigen entrapped by lipids with immunogenic characteristics such as Quil A [25].

Organic polymeric NPs are divided into synthetic and naturally occurring polymers. Examples of the former are polystyrene, and PLGA (dl-Lactic co-glycolic acid), which were reported to be efficacious in delivering and promoting controlled release of growth hormone in monkeys as well as exhibiting adjuvant functions [27]. Examples of naturally occurring polymers forming NPs are pullulan (CHP) [30] as well as nanogels derived from chitosan, both of which have been reported to be highly potent as adjuvants [31]. Chitosan has been used in humans as an excipient in a number of formulations and has shown good tolerability and low toxicity [32].

For self-assembling proteins of bacterial origin, the most important representative is ferritin (Figure 1D). The disadvantages of this system are the low valency of ferritin—with 24 subunits compared to other NPs such as VLPs with 180 subunits—and its small size (<10 nm), which are thought to render the particle less immunogenic; however, relevant pre-clinical results are being reported using such a platform with an excellent immunogenic profile. Studies to develop a vaccine against Influenza H1N1 were reported to be successful [33] and, more recently, the development the Epstein-Barr virus vaccine was reported with strong pre-clinical data [34].

At the moment, VLPs remain as the sole class of NP currently approved for use in humans as vaccines. This is most likely a consequence of the long host-pathogen co-evolution of vertebrates and viruses. The immune system has evolved over time to recognize conserved patterns of viruses that are also present in VLPs, viral vectors and some NPs, inducing a strong protective immune response. However, the range of NPs available and the fast pace of development will likely raise other classes of NPs to a similar status.

## 3. NPs Successfully Used as Vaccines

The first NP-based vaccine licensed for humans dates back to 1981, a VLP-based vaccine against hepatitis B virus (HBV) [38]. The recombinant HBV vaccine became feasible once researchers found sub-viral particles of HBV in the sera of infected subjects. The particles of sub-viral size are spherical assemblies of the HBV surface antigen (HBsAg) with a size of 22 nm; however, the structures are devoid of nucleic acid, and therefore non-infectious [36]. This discovery led to the development of the HBsAg derived from the plasma of infected carriers. Safety concerns regarding the use of human-derived products encouraged the development of second-generation VLPs, produced by recombinant engineering [39,40]. Two decades later, two other VLP-based vaccines were approved for use in humans, targeting human papilloma virus (HPV) with the trade name Cervarix^®^ and Gardasil^®^ (Figure 1B), which are hopefully reducing HPV-induced cancers by 50% [41]. In 2011, a fourth VLP-based vaccine was licensed for use in humans in China, a vaccine against hepatitis E. China remains the sole country so far in which the vaccine has been licensed [37].

**Figure 2:**
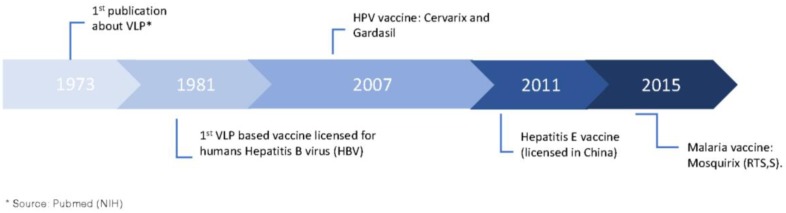
Timeline of the licensing of NP-based vaccines for humans. Five vaccines based on NPs are currently licensed for humans. Of note, all NPs are VLPs.

A fifth VLP-based vaccine against malaria was recently approved for use in humans. The RTS,S/AS01 with the trade name of Mosquirix^TM^, developed by GSK, is a mosaic particle based on the hepatitis B surface antigen (HBsAg) fused to a large part of the *P. falciparum* circumsporozoite antigen (CSP). The vaccine was approved with acceptable profiles of safety and tolerability. The reported efficacy rate was as high as 50% in young children in the endemic area of sub-Saharan Africa, leaving, however, room for improvement [23]. The timeline of development of NP-based vaccines is represented on Figure 2.

## 4. Immunology of NPs

The immune response against NPs is known to elicit both arms of the immune system, innate and adaptive. The following sections will cover the general immune response elicited by those particles and the strategies employed by researchers to manipulate and improve the immune response. 

### 4.1. Fluid Phase Pattern Recognition Molecules

The innate immune system has an important defense line composed of biochemical molecules such as the complement system molecules [42] and pentraxins [43], which circulate through the blood stream in inactive form. Under favorable conditions, these molecules become activated and provide important danger signals to the immune system.

The repetitiveness of viruses and, likewise, NPs promotes the deposition and fixation of the components of the complement cascade and other multimeric proteins. This is a result of the constant but slow deposition of C3b of the classical pathway in foreign surfaces that occurs spontaneously [42]. The repetitive surface of NPs, once covered by C3b, enhances the activity of the C3bBb convertase, amplifying the cascade of the reactions part of the complement [44]. Other factors such as charge, size and the nature of the building blocks of each NP are reported to interact and skew complement activation in a NP-specific manner [28,45]. The activation of the complement system augments the opsonization of antigens, reduces the signaling threshold needed to activate the B cell receptor (BCR) [42], provides co-stimulation to B cells by C3 degradation products and promotes antigen trapping in the germinal center, which contributes to the generation of memory B and long-lived plasma cells [29]. In addition to the activation of B cells, the deposition of complement releases a potent anaphylatoxin C3a and the chemoattractant C5a, which promotes the influx of immune cells to the site and the activation of APCs, boosting the antibody response and overall immune response [46].

The surface of NPs can also be chemically manipulated to improve activation of complement; this approach has been used in an experimental murine model to harness the activation of complement for vaccination purposes. Specifically, poly-hydroxylated NPs such as pluronic-stabilized polypropylene sulfide (PPS) were shown to spontaneously activate complement by improving the deposition and fixation of the C3b component of the alternative pathway [47]. Such NPs were shown to strongly activate and induce maturation of dendritic cells (DCs) when compared to non-hydroxylated polystyrene nanospheres [47]. Others have also reported the differential deposition of complement components on polyethylene glycol-based (PEG) NPs with variable density of PEG and size. Ultimately, the varying degrees of complement deposition and activation impacted the macrophage uptake of the NPs [28].

On the other side of the spectrum, the complement system has been reported as dampening the in vivo effects of liposomes. The deposition of components of the complement system leads to rapid clearance without further immune activation [26].

This branch of the innate immune system has not received much attention as a target of adjuvants and, although poorly explored, the contribution to the immune response against NPs is well established, as viruses and NPs spontaneously activate complement that influences B and T cell responses [42,45,48]. Further studies are necessary to demonstrate the feasibility of modulating and increasingly engaging those molecules for improved vaccination strategies.

### 4.2. Size and Bio-Distribution

The small size of VLPs is an important characteristic and may be viewed as a pathogen-associated structural pattern (PASP). Pathogenic agents such as viruses and bacteria have size distributions of 10 nm to 3 μm [7], while there is almost no self-protein in the fluid of vertebrates that falls within the same size range. Viruses have their size and complexity restricted by the size of their genomes and the small number of proteins that they are capable of encoding [49]. Moreover, the structure of the lymph vessels excludes complexes larger than 500 nm from freely entering the lymphatic system and lymph nodes [47]. This favors the entry of pathogens into lymph nodes, which is where the adaptive immune response is initiated, and it is an important mechanism for directing viral-size pathogens towards lymph nodes where innate cells specialized in dealing with such particles reside. Within lymphoid organs, small particles are not only transported to B cell follicles [50] but also preferentially taken up by DCs and macrophages, but not other cell types [51,52], focusing antigen uptake on the most important cells for the induction of protective immune responses.

Another important biophysical property of particles is charge. Nano-emulsions have been reported to increase the infectivity of phages in vitro due to size distribution and zeta potential, with possible implications for bacterial control in a *S. aureus* infection model [35]. Pre-clinical studies have also demonstrated that by altering the charge in lipid-based particles, the profile of bio-distribution in the tissue and the kinetics of these particles are changed [12]. This is likely a result of charge-dependent interactions with proteins in the serum and the complement deposition that influence the clearance of foreigner particles in vivo [53]. Of note, it is important to consider that when changing the charge, the compositions of lipids are altered, and this could also contribute to the effects observed.

Although size and charge as a PASP and a potent immunogen is a consensus, conflicting reports on the impact of size on the immune response add difficulties to setting an optimal and narrow range of size and charges. This is likely a result of additional immunogenic features of the NPs not controlled in the experiment. This problem and other confounding factors are extensively reviewed elsewhere [54].

### 4.3. DCs and Macrophages

DCs and macrophages are the main APCs, and, hence, targeting these cells enhances and focuses antigen presentation driving strong T cell responses. APCs are not only responsible for presenting the antigens to the relevant lymphocytes, in particular T cells, but they also influence the quality of the T cell responses induced through the composition and levels of cytokines and stimulatory signals generated. Furthermore, engaging T cell receptors (TCRs) of T cells without secondary signals from APCs may trigger tolerogenic pathways, leading to the abrogation of cellular responses [55]. Thus, targeting APCs and modulating their activity represents possibly the most efficacious strategy of inducing cellular responses of a desired nature.

**Figure 3:**
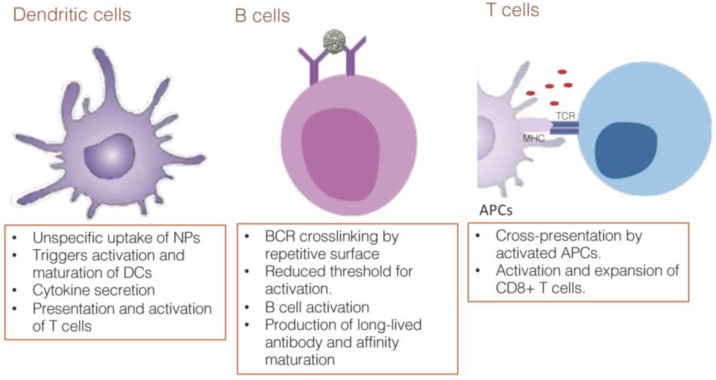
Interaction of NPs and relevant immune cells. APCs such as dendritic cells are the main cells recognizing and driving the immune response against NPs. The unspecific uptake guarantees that those cells will internalize and process most of the pathogens and molecules. The uptake of NPs triggers maturation of DCs and secretion of relevant cytokines that will stimulate other cells, such as T cells, and modulate the immune response. Humoral immune response is favored by the repetitive surface which promotes BCR crosslinking and activation of B cells, leading to activation and production of long-lived antibodies. Cellular and cytotoxic responses are driven by the APCs that internalized the NPs and cross-present the antigens to T cells.

Once APCs, especially DCs, uptake and process the NPs, they go through maturation and reach an activated state (Figure 3). Once activated, these cells are able to present antigens to T cells and initiate the adaptive immune response. It has been shown that DCs are able to present VLP-bound antigens both through the classical major histocompatibility complex II (MHC) pathway and also cross-present to charge MHC I molecules. This leads to both Thelper (Th) and CD8^+^ T cell responses even in the absence of infection [56].

An additional important feature of NPs is that they can transport adjuvants as cargo for delivery to endosomal compartments of APCs, engaging important pattern recognition receptors (PRRs), such as TLRs that skew and trigger stronger immune responses. Many VLPs and all viral vectors naturally encapsulate nucleic acid inside a protein shell, and other NPs can easily be assembled around the ISS and promote a similar adjuvant effect [6,32,57]. For VLPs, the nucleic acid is non-infectious, but is usually necessary for virus assembly and appropriate folding; however, the NA can be removed and/or replaced for downstream applications [58]. Generally, the foreign nucleic acid packaged in NPs is released within endosomal compartments of APCs after particle uptake and protein degradation, activating TLR3 in the presence of dsRNA, TLR7/8 with ssRNA [59] and TLR9 with non-methylated DNA rich in CG areas, the CpG islands [60]. The activation of those pathways leads to the production of Th1-associated cytokines such as IFNα, TNFα and IL-1β by DCs and CCL5 and TNFα by macrophages in a TLR9-dependent manner [61]. It also activate interferon-stimulated genes (ISG) related to antiviral control as well as the production of the key cytokine IL-12 [62]. Other cytosolic sensors such as RIG-I and STING have been implicated in the response against VLPs in a few studies. One study showed STING activation by membrane disturbance caused by the entry of viral vectors, VLPs and liposomes [63]. The use of 5′-triphosphate–containing RNA, a RIG-I agonist, combined with an influenza-derived VLP was capable of increasing antibody titers and protecting mice against a lethal challenge with H5N1 [64]. Another study suggested an involvement of endogenous retrovirus-derived nucleic acids that would activate RIG-I and cGAS [65] upon VLP recognition by B cells.

The aforementioned immunomodulatory properties of NPs have been used for improving vaccine formulation and nucleic acids, and other TLR ligands have been employed as adjuvants in order to harness the anti-viral defenses of the body [66]. CpG is one of the most extensively studied TLR ligands used as an adjuvant in pre-clinical models. Several papers have shown CpG as a strong inducer of protective T cell responses in different disease models, such as lymphocytic choriomeningitis virus (LCMV) and cancer [67,68,69,70]. Another example of the usage of TLR ligands is the TLR4 ligand monophosphoryl lipid A (MPLTM), used as an adjuvant in the commercial Cervarix vaccine against HPV [71]. MPL^TM^ is a chemically modified derivative of lipopolysaccharide (LPS) and has been shown to induce a stronger and prolonged response when compared to the VLP alone. A RNA-lipoplex (RNA-LPX) system was also used as an adjuvant in cancer models and a phase I dose-escalation trial in melanoma patients is showing promising results for patient-specific vaccines [12].

### 4.4. B Cell Responses

NPs are usually made up of repetitive subunits. T = 3 icosahedral VLPs, for example, are built of 180 copies of a single protein in quasi-equivalent conformations [72]. Evolution of the host-pathogen interaction rendered B cells highly responsive to such particles. Repetitive surfaces with antigenic determinants with a density of 12–16 and that are closely spaced—the optimal range being considered 10 nm—[73] promote crosslinking of the BCR, which constitutes an activation signal for B cells and eliminates the need for T cell help (Figure 3). As discussed earlier, the deposition of complement on the surface increases the interaction of VLPs and follicular dendritic cells, driving the germinal center reaction. In addition, complement degradation products on NPs engage CD21 on B cells, enhancing B cell activation as well as the induction of long-lived plasma cells and class switching.

It has been shown that TLR ligands packaged inside NPs such as VLPs or liposomes greatly improve IgG responses against the model antigen TCR when compared to soluble CpG mixed with the antigen. An interesting finding of this paper relates to the synergic role of DCs and B cells. Using tissue-specific MyD88 knockouts, the impact of linking versus mixing antigens with TLR ligands was compared for VLPs and soluble OVA. For soluble protein, TLR recognition and signaling on DCs was more important for antibody responses when compared to TLR signaling in B cells. This observation was explained by the activation of T helper cells by activated DCs, providing the signals necessary to activate B cells. In contrast, for VLPs packaging CpG and presenting antigens, the TLR signaling in DCs and subsequent T cell-mediated help seemed no longer necessary, as TLR-signaling on B cells were more important for the response [74,75].

The importance of TLR ligands present in NPs for antibody responses largely depends on the nature of the antigen and the interplay with BCR activation [76]. It was reported that highly repetitive antigens are able to induce high IgG titers even in the absence of MyD88 in response to TLR stimulation [77]. However, in other models where the antigen provides weaker BCR ligation, the absence of the TLR ligand resulted in a 30-fold reduction in IgG titers [74]. Regarding the quality of the antibody response, it is well established that TLR ligands impact isotype switching. Activation of TLR9 in B cells is shown to favor the production of IgG2a in mice and IgG1 in humans over other subtypes in a T-bet–dependent manner [77,78].

Another important aspect of the use of VLPs with packaged TLR7/8 and TLR9 ligands is the increased induction of antigen-specific germinal centers (GC), which boosts the levels, affinity and isotype switching of antibodies. A 10-fold increase in the formation of antigen-specific GC has been reported upon immunization with antigens coupled to a TLR ligand compared to free antigen [74].

### 4.5. T Cell Responses

To date, most of the vaccines produced and licensed for use in humans confer protection through the induction of antibodies. This approach has proven successful in many cases; however, it has consistently failed in many diseases that represent a great burden to public health systems such as malaria and HIV. It is likely that for such diseases, humoral responses alone are not enough to confer protection, and it is necessary to engage other arms of the immune system such as T cells. Great efforts have therefore been made towards the development of adjuvants focusing on DC and T cell activation (Figure 3).

Although NPs are mostly known as good inducers of antibody responses, they also hold desirable characteristics that can be explored to induce a cellular response. As discussed above, NPs have the right size to freely drain to lymph nodes from peripheral areas to encounter lymph node–resident APCs. As only lymph node–resident CD8^+^ DCs can cross-present antigen, their adjusted size can be used to induce cytotoxic T cell responses even in the absence of infection [79]. In addition, NPs are also taken up by skin- or muscle-resident DCs, which causes DC maturation, leading to the upregulation of the chemokine receptor CCR7, a homing receptor that causes DCs to migrate to lymph nodes [80], where they arrive as fully mature DCs charged for T cell priming. Hence, NPs are presented to T cells in an orchestrated way by a large number of different professional APCs, which increases the chance of robust T cell activation. The ability of NPs to package RNA or DNA is a key feature in this context, as this leads to concomitant activation of peptide-presenting DCs, further driving T cell responses.

Using VLPs, it has been shown that CpGs augment the induction of antigen-specific CD8^+^ T cells when compared to the same VLP containing RNA or no TLR ligand [10], and most importantly, they are able to reduce the toxic effects of CpG in murine models [68]. The increased expansion of specific CD8^+^ T cells was correlated with anti-viral protection in the same study. There have also been a number of studies using CpG-loaded VLPs for induction of melanoma-specific T cell responses in humans. A combination with Imiquimod, a ligand for TLR7, was particularly effective [81,82].

For influenza vaccines, the induction of T cells responses holds promise for the production of a universal influenza vaccine. Although antibody-based vaccines are protective, they usually are serotype specific. A recent study demonstrated that, utilizing regimens favoring T cell response, it was possible to protect in a mouse-adapted influenza model against homosubtypic and heterosubtypic influenza, as the T cell response generated target conserved epitopes among those strains [83,84].

Another approach to improve T cell responses would also include adjusting vaccine schedules. It has been shown that using escalating doses that reach a peak, followed by a decrease, is more efficient in inducing T cell responses when compared to a schedule with a fixed dosage [85].

In the opposite trend, NPs are being modified to shut down cytotoxic T cells instead. This approach aims to induce immune-tolerance by activating Tregs against specific auto antigens involved with auto-immune diseases, for example. A well-described target is the aryl hydrocarbon receptor present in DCs [86], a ligand-activated transcription factor that leads to tolerogenic responses via FoxP3^+^ T cells. Along the same lines, nanoparticles of poly(lactide-co-glycolide) bearing an antigen related to encephalomyelitis were able to ameliorate the disease by inducing antigen-specific Tregs and leading to an anergic response. This was achieved by directing the uptake of the nanoparticle to a specific population of macrophages bearing the scavenger receptor MARCO [87].

Whether the goal is activation or silencing of cytotoxic T cells, it has become clear that the primary target should be the APC, not the T cell itself.

## 5. NPs for Chronic Non-Communicable Diseases

The immune system has evolved several checkpoints to avoid self-recognition and auto-immunity. This is an important feature of the immune system and it is key to balance the capacity to recognize and clear infections. Several diseases, however, such as Parkinson′s and Alzheimer′s diseases, are related to the aggregation of self-proteins, and a large number of chronic diseases, such as rheumatoid arthritis and psoriasis, are caused by the excessive production of inflammatory cytokines. The immune system is often unable to tackle these diseases as it has learned not to respond to the “self”. NPs, however, are capable of bypassing tolerance against self-proteins by adding a viral fingerprint to those proteins [88]. This finding opened new possibilities to employ NPs not only as vaccines against infectious diseases but also against chronic and auto-immune diseases. For psoriasis, it is now well established that interleukin IL-17 is involved in the formation of the skin lesions, a notion recently confirmed in humans by the success of monoclonal antibodies against IL-17A or the receptor IL-17RA in controlling and reversing the skin disease [89]. These treatments are now on the market or in phase III trials with great safety profiles and they are highly efficacious [90]. Although successful, employing monoclonal antibodies against chronic diseases can be a costly endeavor, and vaccination stands as a more cost-effective alternative. It has been shown that a vaccine employing a VLP coupled to IL-17 was capable of ameliorating the symptoms of auto-immune disease models of encephalitis and arthritis [15].

Another example of vaccines targeting self-antigens is the CAD106 vaccine against Alzheimer′s. The rationale behind this vaccine is to induce antibodies against the self-protein Aβ implicated in the pathology of Alzheimer’s. The vaccine has reached phase III now, with acceptable safety levels and a confirmed capacity to induce long-lasting antibodies [17].

## 6. Use of NPs as Anti-Viral and Delivery Systems

Due to the diversity of materials from which NPs can be built, different particles can be employed in a range of applications that are not limited to the field of vaccines.

The use of NPs has been reported with functionalized surfaces to harbor anti-viral properties, by interacting with the hydrophobic regions of the envelope of the HIV virus and reducing the capacity of the infection of the virus. The treatment was able to reduce the transduction ability of viruses in 50% [91]. Although rather preliminary, this study provides a new range of application for NPs.

A promising field that also relates to vaccines is using NPs as a cargo of immunogens that can modulate the immune system. The advantages of packing TLR ligands inside VLPs and other NPs, as mentioned earlier, are already well established, and the response generated against heterologous antigens coupled to VLPs with CpG is remarkably stronger in terms of the expansion of specific CD8^+^ T cells and the production of cytokines [68,70]. Along the same lines, different groups have been attempting to pack other immune modulators, such as small interference RNA (siRNA), in addition to functionalization of the surface of the particles which allows the targeted delivery of siRNA to specific cell types [57]. Studies in mice employing the concomitant use of CpG and siRNA encapsulated by liposomes targeting the anti-inflammatory cytokine IL-10 in a vaccine regime against B cell lymphoma [92] and melanoma [93] demonstrated the ability to modulate the Th1:Th2 balance in order to improve anti-tumor immunity.

Beside the promising results so far, this system stands as an interesting platform since it is able to modulate cytokine production without any long-lasting effects. This is not only important for clinical applications, but it will very likely provide an important tool for further understanding the intricate dynamics of cytokines and the immune system.

## 7. Final Remarks

The use of NPs for vaccine development holds enormous promise not only for the vaccine field but also as a valuable tool to study the ins and outs of the immune system. To observe and learn how the immune system of vertebrates, especially mammals, evolves in response to viruses and other pathogens can provide valuable clues that will enable researchers to rationally manipulate the immune response and tailor better vaccines. The growing number of NPs with different immunomodulatory and adjuvant characteristics will provide the tools necessary to develop more efficient vaccines, both prophylactic and therapeutic, aiming to tackle infectious, autoimmune and chronic diseases.

Currently, great efforts are being made towards understanding and modulating the immune response raised by NPs. To this end, basic and clinical research shall elucidate the mechanisms and the optimal combinations and regimens to better mimic pathogens and to induce protective responses and memory.

## Figures and Tables

**Table 1:** General classes of nanoparticles (NPs).

Particle	Characteristics and Mechanisms	Size	Commercial Name	Ref.
Emulsions	Oil in water emulsions composed of a solvent and a surfactant. Vaccine adjuvant, leads to recruitment of immune cells and induction of Th1 response.	50–600 nm	MF59™, Montanide™	[23,24]
Inorganic NPs	Rigid structure and controlable synthesis. Non-biodegradable.	0.8–200 nm	AuNPs (Gold), Fulleren	[21]
ISCOM	Immune-stimulating complex.“Cage-like” particles. Popular ISCOMs are made of saponin, cholesterol and phospholipds.	40 nm	ISCOM, ISCOMATRIX	[25]
Lipid-based NPs	Biodegradable lipidic NPs such as liposomes, micelles and solid lipids nanoparticle. Encapsulation of antigens with controlled release.	200–1000	DOTAP	[5,26]
Polymeric NPs	Synthetic polymers. Allows controled release of antigens or drugs. Biodegradable.	Variable	PLG, PEG, polystyrene	[27,28,29]
Carbohydrates	Natural polysaccharide. Shape and size are easily manipulated with impact on the profile of the immune response. Biodegradable.	Variable	Pullulan, Advaxa™ (Inulin)	[30,31,32,33]
Self-assembled proteins	Self-assemblying proteins that fold into complex quartenary structure.	10–40 nm	Ferritin, MVP.	[34]
Viral Vectors	Efficient gene transfer for transiente of stable expression. Induce robust CTL responses. Good safety and tolerability profile from clinical trials in humans.	Variable	MVA, Adeno	[35]
VLPs	Self-assembled viral capsides devoided of infectious nucleic acid. Confers viral fingerprint to displayed antigens.	15–50 nm	Gardasil, Cervarix.	[36,37]

VLPs: Virus-Like Particles; CTL: Cytotoxic T Lymphocytes; NPs: Nanoparticles; ISCOMs: Immune-stimulating complex; Th1: T helper 1.
